# Comparison of Serum Apolipoprotein Levels of Diabetic Children and Healthy Children with or without Diabetic Parents

**DOI:** 10.1155/2012/490381

**Published:** 2012-07-03

**Authors:** Mohammad Hashemi, Mohammad Saadat, Mohaddeseh Behjati, Roya Kelishadi

**Affiliations:** ^1^Cardiology Department, Faculty of Medicine, Isfahan University of Medical Sciences, Isfahan, Iran; ^2^Pediatrics Department, Faculty of Medicine and Child Growth & Development Research Center, Isfahan University of Medical Sciences, Isfahan, Iran

## Abstract

*Introduction*. The association of diabetes and atherosclerosis with disorders of lipids and lipoproteins, notably high apolipoprotein B (apoB) and low apolipoprotein A1(apoA1) is well established. Because of the beginning of the atherosclerosis' process from early life, in this study, the plasma levels of apoA1 and apoB were compared in diabetic children with type I diabetes mellitus(DM), healthy children with diabetic parents (HDPs),and healthy children with nondiabetic parents (HNDPs). *Methods*. This case-control study was conducted among 90 children aged 9–18 years. Serum levels of apoA and apoB were compared among 30 diabetic children (DM), 30 healthy children with diabetic parents (HDPs), and 30 healthy children with nondiabetic parents (HNDP). *Results*. The mean serum apoA1 was higher in DM (153 ± 69 mg/dL) followed by HNDPs (138 ± 58 mg/dL) and HDPs (128 ± 56 mg/dl), but the difference was not statistically significant. The mean apoB value in HNDPs was significantly lower than DM and HDPs (90 ± 21 mg/dL versus 127 ± 47 and 128 ± 38 mg/dL, *P* < 0.05, respectively). The mean apoB levels in DM (127 ± 47 mg/dl) and HDP (128 ± 38 mg/dL) were not statistically significantly different (*P* > 0.05). *Conclusions*. Diabetic children and healthy children with diabetic parent(s) are at higher risk of dyslipidemia and atherosclerosis. Thus for primordial and primary prevention of atherosclerosis, we suggest screening these children for low plasma apoA1 and high plasma apoB levels.

## 1. Introduction

Atherosclerosis is the most common cause of death in world [1]. Fatty streaks are the first atherosclerotic lesions and are developed since childhood. These lesions are also seen in a child of hyperlipidemic mothers. One of the risk factors of atherosclerotic cardiovascular disease is diabetes mellitus (DM) and is associated with worse prognosis in patients with diagnosed coronary artery disease. The risk of myocardial infarction (MI) in nondiabetic patients with and without previous history of MI is similar to diabetic patients without previous history of MI [2]. Then, treatment of risk factors in diabetic patients without previous history of MI should be the same with nondiabetic patients with previous history of MI [2].

The most important cause of atherosclerosis in diabetic patient's is diabetic dyslipidemia [2]. Diabetic dyslipidemia includes hypertriglyceridemia, increased LDL, decreased HDL, high apoB and low apoA1 [3]. High apoB, and low apoA1 levels seen in type II DM are associated with increased risk of atherosclerotic cardiovascular diseases [3]. Increased plasma glycosylated lipoproteins are also seen in diabetic patients [4]. Diabetic dyslipidemia is associated with quantitative and qualitative changes in plasma lipids and lipoproteins [3]. In type I DM mild changes in shape and content of atherogenic apoB occur that predispose patients to increased risk of atherosclerosis [5]. In these patients, classic lipid profile may be normal but patient is at increased risk of atherosclerosis [5]. Measurement of apoA1 and apoB in diabetic patients may be helpful in diabetic patients at risk of cardiovascular diseases. Benefits of measurement of apoA1 and apoB in diabetic children or healthy children with diabetic parents are not determined. These patients are at increased risk of cardiovascular diseases. So, In this study, plasma levels of apoA1 and apoB were compared in diabetic children with type I diabetes mellitus (DM), healthy children with nondiabetic parents (HNDPs), and healthy children with diabetic parents (HDPs).

## 2. Methods and Materials

 In this case-control study, three groups of children (9–18 years old) were selected by simple random sampling: 30 healthy children (FBS < 110 mg/dL) without diabetic parents (HNDPs), 30 healthy children with diabetic parents (one or both) (HDPs), and 30 children with diagnosis of diabetes (DM) and under the treatment with Insulin for at least 2 year, both males and females. Exclusion criteria were poor control diabetes with HbA1C > 8.5%, history of lipodystrophy, glycogen storage disease, chronic kidney disease, nephritic syndrome, glomerulonephritis, chronic liver disease, physical inactivity, obesity, high-fat diet, alcohol consumption, hypothyroidism, treatment with corticosteroid, retinoid drugs, immune suppressive drugs, growth hormone, hydrochlorothiazide, beta-blockers, and steroid hormones. Serum levels of apoA (reference limit: 94–199 mg/dL) and apoB (reference limit: 60–133 mg/dL) were measured by Kits with 95% sensitivity (Parsazmon, Iran) using immunoturbidimetery method.

## 3. Results

**Figure 1:**
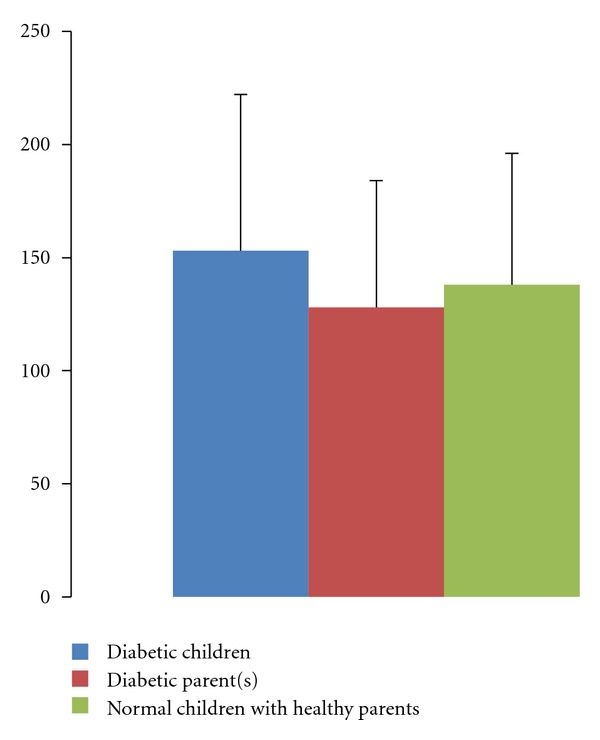
Comparison between serum apoA1 level in diabetic children, normal children with healthy parent(s), and healthy children with nondiabetic parents.

**Figure 2:**
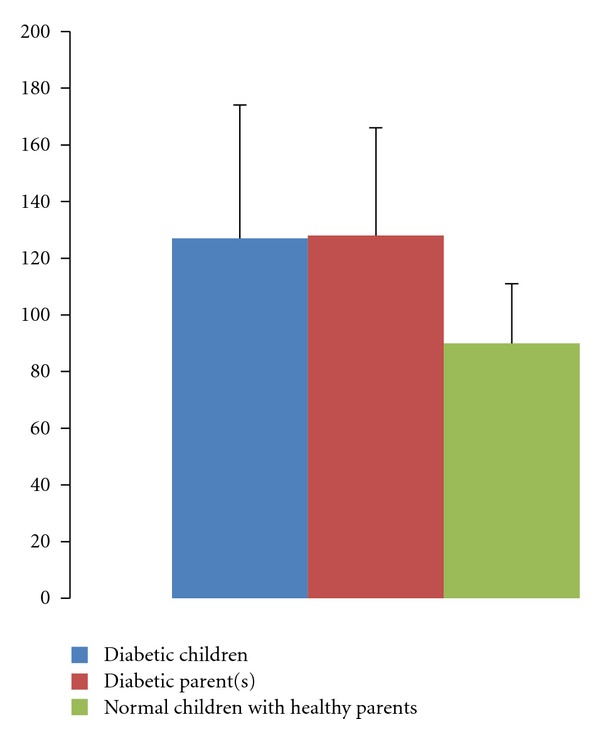
Comparison between serum apoB level in diabetic children, normal children with healthy parent(s), and healthy children with nondiabetic parents.

The mean value of apoA1 in DM was 138 ± 58 mg/dL lower than healthy children with mean values of 153 ± 69 mg/dL, but this difference was not statistically significant (*P* > 0.05). The mean value of apoA1 in healthy children with nondiabetic parent(s) 128 ± 56 mg/dL was higher than healthy children with diabetic parent(s), but this difference was not statistically significant (*P* < 0.05). The mean value of apoA1 in diabetic children was higher than healthy children with diabetic parent(s), but this difference was not statistically significant (*P* > 0.05) (Table 1). Mean apoB value in healthy children with nondiabetic parent(s) was 90 ± 21 mg/dL which was significantly lower than diabetic children with mean values of 127 ± 47 mg/dL (*P* < 0.05). The mean apoB levels in healthy children with nondiabetic parent(s) were 90 ± 21 mg/dL which was significantly lower than children with diabetic parent(s) with the mean level of 128 ± 38 mg/dL, but this difference was not statistically significant (*P* > 0.05) (Table 2). The Mean levels values of apoA1 and apoB in healthy children with nondiabetic parents, healthy children with diabetic parents, and diabetic children are shown in Figures 1 and 2, respectively.

## 4. Discussion

The most common cause of death in diabetic is atherosclerotic cardiovascular disease [2]. Multiple cardiovascular risk factors like hypertension, obesity, and cigarette smoking are common in both type I and II DM, but diabetic dyslipidemia is the most important cause of increased risk of atherosclerosis in diabetic patients [2]. Increase of apoB in diabetic patients demonstrates increased plasma atherogenic lipids [3]. Decreased HLD is also associated with decreased athero-protective apoA levels [3]. Fasting and postprandial apoB in diabetic patients is higher than healthy persons [6]. Improvement in control of type II DM was associated with decreased level of postprandial chylomicrons and lower transport of cholesterol to the apoB lipoproteins [7]. Plasma apoA1 and apoB levels are stronger predictors of premature atherosclerosis than other plasma lipoproteins [8]. Apoproteins are important risk factors for coronary artery disease in future for children with greater risk of atherosclerosis [9]. In one study on diabetic children, increased plasma apoB and dense LDL were associated with poor diabetic control [10].

In our study, in diabetic children apoB levels were higher than healthy children with and without diabetic parent(s). Plasma levels of apoA in diabetic patients were nonsignificantly higher than healthy children. In children with diabetic parent(s) plasma levels of apoA was nonsignificantly lower than healthy children with nondiabetic parent(s). In these children, plasma levels of apoB were nonsignificantly higher than healthy children with nondiabetic parent(s). Then, healthy children with diabetic parent(s) are at greater risk of atherosclerosis and screening for plasma apoA and apoB levels may be helpful in these children. Given the role of healthy lifestyle in prevention and control of non-communicable diseases [11], the barriers to healthy habits should be determined [12], and families should be encouraged for screening and preventive programs.

## 5. Conclusions

For primordial and primary prevention of atherosclerotic diseases, we suggest screening diabetic children and healthy children with diabetic parents for low plasma apoA1 and high plasma apoB.

## Figures and Tables

**Table 1:** Comparison of apoA1 levels in diabetic children, healthy children with diabetic parent(s), and healthy children with nondiabetic parent(s).

Groups studied	Apolipoprot-in in A1 (mg/dL) mean ± SD		*P* value	
		Diabetic children	Healthy children with diabetic parent	Healthy children with nondiabetic parent
Diabetic children	153 ± 69	—	0.27	0.62
Healthy children with diabetic parent	128 ± 56	0.27	—	0.80
Healthy children with nondiabetic parent	138 ± 58	0.62	0.80	—

**Table 2:** Comparison of apoB levels in diabetic children, healthy children with diabetic parent(s), and healthy children with nondiabetic parent(s).

Groups studied	Apolipoprot-in B (mg/dL) mean ± SD		*P* value	
		Diabetic children	Healthy children with diabetic parent	Healthy children with nondiabetic parent
Diabetic children	127 ± 47	—	1.00	0.001
Healthy children with diabetic parent	128 ± 38	1.00	—	<0.0001
Healthy children with nondiabetic parent	90 ± 21	0.001	<0.0001	—
